# The Sonic Hedgehog Pathway Modulates Survival, Proliferation, and Differentiation of Neural Progenitor Cells under Inflammatory Stress In Vitro

**DOI:** 10.3390/cells11040736

**Published:** 2022-02-20

**Authors:** Mohamed Tail, Hao Zhang, Guoli Zheng, Maryam Hatami, Thomas Skutella, Andreas Unterberg, Klaus Zweckberger, Alexander Younsi

**Affiliations:** 1 Department of Neurosurgery, University Hospital Heidelberg, 69120 Heidelberg, Germany; mohamed.tail@med.uni-heidelberg.de (M.T.); zhanghaoayfy523@gmail.com (H.Z.); guoli.zheng@med.uni-heidelberg.de (G.Z.); andreas.unterberg@med.uni-heidelberg.de (A.U.); k.zweckberger@klinikum-braunschweig.de (K.Z.); 2 Department of Neuroanatomy, Institute for Anatomy and Cell Biology, University of Heidelberg, 69120 Heidelberg, Germany; maryam.hatami@uni-heidelberg.de (M.H.); thomas.skutella@uni-heidelberg.de (T.S.)

**Keywords:** Shh, NPC, spinal cord injury, LPS, neuroinflammation, neuroregeneration, Cyclopamine, Ki-67, in-vitro, differentiation

## Abstract

The Sonic Hedgehog protein (Shh) has been extensively researched since its discovery in 1980. Its crucial role in early neurogenesis and endogenous stem cells of mature brains, as well as its recently described neuroprotective features, implicate further important effects on neuronal homeostasis. Here, we investigate its potential role in the survival, proliferation, and differentiation of neural precursors cells (NPCs) under inflammatory stress as a potential adjunct for NPC-transplantation strategies in spinal cord injury (SCI) treatment. To this end, we simulated an inflammatory environment in vitro using lipopolysaccharide (LPS) and induced the Shh-pathway using recombinant Shh or blocked it using Cyclopamine, a potent Smo inhibitor. We found that Shh mediates the proliferation and neuronal differentiation potential of NPCs in vitro, even in an inflammatory stress environment mimicking the subacute phase after SCI. At the same time, our results indicate that a reduction of the Shh-pathway activation by blockage with Cyclopamine is associated with reduced NPC-survival, reduced neuronal differentiation and increased astroglial differentiation. Shh might thus, play a role in endogenous NPC-mediated neuroregeneration or even be a potent conjunct to NPC-based therapies in the inflammatory environment after SCI.

## 1. Introduction

The Hedgehog (Hh) pathway is one of the critical inter-cell communication pathways during the embryonic development of vertebrates. Sonic Hedgehog (Shh) thereby exerts essential functions in limb development [1] and, among other morphogens, is well known for its crucial effects on the premature and adult central nervous system (CNS) [2,3,4]. Similar to Wingless-type MMTV Integration Site Family Member 2 (Wnt2) and other growth factors, Shh has diverging effects depending on its dosage and timing of exposure [5], especially on Mesenchymal Stem Cells (MSCs) and Neural Precursor Cells (NPCs) [3,6,7]. For NPCs, their effects on the formation, proliferation, and differentiation are well described during the organogenesis of the dorsoventral pattern of the neural tube and cortex [8,9].

However, even though its influence diminishes after embryogenesis, Shh is still secreted in stem cell-rich areas in the adult mammalian brain [3]. More recent publications observed that Shh affects stem cell niches in the postnatal and adult brain’s subventricular zone (SVZ) by modulating precursor cells and controlling their proliferation [4]. Such SVZ-derived NPCs with the ability to migrate over long distances might play an essential role in brain tissue homeostasis and endogenous neuroregeneration after injury to the CNS [10]. Moreover, they are already used for experimental stem cell transplantation strategies in the context of spinal cord injuries (SCI), with the goal to improve neuroregeneration by exogenous cell grafts [11]. Similarly, Shh has been directly associated with protection and repair in adult neurological diseases [12,13].

The question thus arises whether Shh is involved in neuroprotective and neuroregenerative mechanisms of the CNS as a response to harmful conditions and injuries by its effects on NPCs. However, because it has been discovered that the disruption of the Shh pathway plays an important role in the tumorigenesis of various tissues, such as the pancreas, the bladder, or even the brain, a substantial part of research associated with Shh has been dedicated to understanding its protooncological characteristics [14,15,16]. Consequently, only a few publications so far tried to shed light on the potential of Shh to modulate the conduct of NPCs in response to injury or stress.

In consideration of the very limited neuroregeneration after SCI, the modulation of endogenous or exogenous NPCs by Shh might be an interesting prospect for future treatments [17,18,19]. Thus, in our current study, we assessed the effects of Shh on the survival, proliferation, and differentiation of NPCs in vitro under inflammatory stress, mimicking the hostile environment of the injured spinal cord.

## 2. Materials and Methods

### 2.1. Isolation and Culture of Neuronal Precursors Cells (NPCs)

NPCs were isolated from the SVZ of 2-week-old (P14) embryos of green fluorescent protein (GFP) expressing transgenic Wistar rats (Rat Resource & Research Center, University of Missouri, Columbia, MO, USA; strain F344-Tg(UBC-EGFP)F455Rrrrc). SVZ tissue pieces free from meninges were obtained and washed in 2 mL cold phosphate-buffered saline (PBS; without CaCl_2_ and MgCl_2_; Thermo Fisher, Waltham, MA, USA). After removing the buffer, 1.5 mL 0.05% trypsin/ethylenediaminetetraacetic acid with 0.2% deoxyribonuclease I (Thermo Fisher, Waltham, MA, USA) was added per 10 tissue pieces. We incubated the suspension at 37 °C for 5 min before the enzymatic activity was inhibited by adding 10% fetal bovine serum (FBS; Thermo Fisher, Waltham, MA, USA). Additionally, the tissue was mechanically dissociated into a cell suspension with a fire-polished pipette before centrifuging for 6 min. Eventually, NPCs were plated in poly-L-ornithine-laminin-coated tissue culture plates at a density of 1.5 × 10 ^4^ cells/cm^2^ in 1.5 mL growth medium (Dulbecco’s Modified Eagle’s Medium/F12 with sodium bicarbonate and l-glutamine (Thermo Fisher, Waltham, MA, USA), 1% penicillin/streptomycin, 1 × N2 supplement (both Gibco, Life Technologies, Carlsbad, CA, USA), 20 ng/mL bFGF, and 10 ng/mL EGF (both Sigma-Aldrich, Burlington, MA, USA). Cells were then incubated in a humidified incubator at 37 °C with 5% CO_2_. When reaching a confluency of 80–90%, NPCs were split at 1:3 and their viability, as well as their stem cell characteristics and tripotential differentiation capacities into all three neural cell types, were successfully assessed before further use (data not shown). All in vitro experiments were performed with NPCs at passage four (P4). All experimental protocols were approved by the Animal Care Committee of the federal government of Baden-Württemberg, Germany.

### 2.2. Treatment Characteristics and Study Design

**Figure 1:**
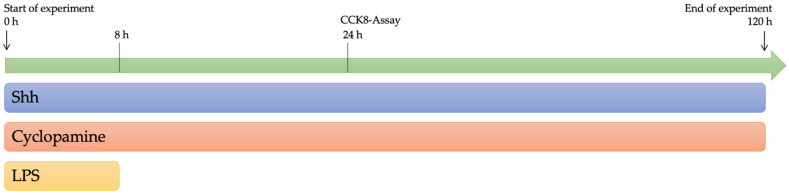
Study design timeline. In the LPS only, the Shh+LPS and the Cyclopamine+LPS groups, exposition of the NPCs with LPS was stopped by medium change after 8 h. The exposition of the NPCs with Shh and Cyclopamine was sustained for either 24 h (CCK8-Assay) or 120 h (immunofluorescence staining).

To assess the effects of Shh on the NPCs, they were cultured on 96-well plates and we either added Shh as a treatment directly (20 nM Shh; R&D Systems, Minnesota, MN, USA) or blocked the Shh pathway with Cyclopamine (20 µM Cyclopamine hydrate; Sigma-Aldrich, Burlington, MA, USA). Furthermore, the cells were either cultured under normal conditions in the above-mentioned growth medium or with the addition of Lipopolysaccharide from *E. coli* (20 µg/mL LPS; Sigma-Aldrich, Burlington, MA, USA) for the first 8 h to simulate an inflammatory environment upon binding to Toll-Like-Receptor 4 (TLR4) like the postinjury environment in the CNS. The medium was changed after 8 h and every day after that until the end of the experiment (Figure 1).

These treatment regimens resulted in the following six treatment groups: Shh only (20 nM Shh), Cyclopamine only (20 µM Cyclopamine hydrate), LPS only (20 µg/mL Lipopolysaccharide from *E. coli*), Shh+LPS, Cyclopamine+LPS and untreated control (growth medium) (Table 1).

### 2.3. Cell-Count-Kit 8 (CCK-8) Viable Cell Quantification

To assess the survival of NPCs after the respective treatment in all groups, we used a ready-to-use Cell-Count-Kit 8 (CCK-8) (Sigma-Aldrich, Burlington, MA, USA). In short, the CCK-8 viable cell quantification is based on the tetrazolium salt WST-8 which is reduced to a less water-soluble formazan dye through intracellular enzymatic reactions or direct NADH or NADPH interaction, indirectly measuring cell death [20]. According to the manufacturer’s protocol, CCK-8 was added to the NPCs on 96-well plates in growth medium 24 h after the respective treatment had been initiated and the absorbance of the resulting formazan dye was measured with an ELISA microplate reader (Sunrise XFLUOR4; Tecan, Männedorf, Switzerland) after incubation for 2 h at 450 nm. For every treatment group, six repetitions of the CCK-8 viable cell quantification were performed, and the results were averaged. The results of the respective treatment groups were then divided by the results of the control group, and therefore, normalized to the values of the control group (presented as %).

### 2.4. Immunofluorescence Staining and Imaging Analysis

To understand the role of the Shh-pathway in the differentiation of NPCs in an inflammatory setting, we used immunofluorescence staining to assess and semi-automatically quantify different evolved cell types five days (120 h) after the respective treatment had been initiated. To this end, NPCs on 96-well plates were fixed with 4% paraformaldehyde for 30 min and washed three times with PBS. The cells were then subjected to the following immunofluorescence staining protocol: PBS was replaced with a blocking solution containing 0.3% Triton-X100, 5% milk powder, and 1% bovine serum albumin (all Sigma-Aldrich, Burlington, MA, USA) and incubated for one hour at room temperature. Next, the following primary antibodies diluted in the above-mentioned blocking solution were added to the NPCs at 4 °C overnight:

Anti-Nestin (1:400; Millipore, Billerica, MA, USA) for undifferentiated NPCs, anti-NeuN (1:500; Millipore, Billerica, MA, USA) for neurons, anti-Olig2 (1:50; Abcam, Cambridge, UK) for oligodendrocytes, anti-GFAP (1:250; Abcam, Cambridge, UK) for astrocytes and anti-Ki-67 (1:250; Abcam, Cambridge, UK) as a proliferation marker.

The cells were then washed three times with PBS and subjected to incubation with the secondary antibodies diluted in blocking solution without Triton-X100 for 1 h at room temperature. The following secondary antibodies were used:

Alexa Fluor 557 donkey anti-mouse (1:400; R&D Systems, Minnesota, MN, USA), Alexa Fluor 647 donkey anti-rabbit (1:400; Abcam, Cambridge, UK), and Alexa Fluor 405 donkey anti-goat (1:400; Abcam, Cambridge, UK).

A confocal laser scanning microscope (LSM 700; Carl-Zeiss, Jena, Germany) was used to obtain images at 10× magnification in the 8-bit-format with the tile scan function (speed of six, gain of 800). Four wavelength channels (Alexa Fluor-405 nm, GFP-488 nm, Alexa Fluor 568 nm, Alexa Fluor 647 nm) were used.

For the quantitative assessment of NPC-differentiation (NeuN^+^/GFP^+^ cells and Olig2^+^/GFP^+^ cells) and NPC-proliferation (Ki-67^+^/ GFP^+^ cells) we used a semi-automatic algorithm for ImageJ2 (National Institute of Health, Bethesda, MD, USA) as previously described [13,21]. Briefly, the images were split into single channels, a Gaussian-filter (Sigma: 10.00) was applied to reduce background noise, and the IsoData-thresholding algorithm was used to transform a selected region of interest (ROI) with 10.25 mm^2^ at the center of each well into a binary image. Positive-labeled cells with signals above specific thresholds were counted in the selected ROI with the “Analyze Particles” function. Next, binary images were recombined using the “Image Calculator” function, and the co-stained cells within the same ROI were counted. To avoid including artifacts, only structures with an area of 10–2000 µm^2^ were considered. The number of the respective co-stained cells/ROI was then divided by the number of GFP^+^ cells/ROI in each well and thus given as a percentage of GFP^+^ cells.

For the quantitative assessment of astrogliosis (GFAP) and undifferentiated NPCs (Nestin), the immunointensity of the respective antibody was quantified. To this end, images were split into single channels with the ImageJ2 software, ROIs in size of 10.25 mm^2^ were placed at the center of the respective well, and the “Measure” function was used to output the respective immunointensity (pixel intensity(pi)/mm^2^). The immunointensity was then divided by the number of GFP^+^ cells/ROI in each well (see above) and results are, thus, given as the respective immunointensity/GFP^+^ cells.

For every treatment group, three repetitions of the respective immunofluorescence staining were performed, and the results were averaged.

### 2.5. Western Blot

To evaluate endogenous Shh production and secretion in NPCs we performed a Western Blot analysis of both cell lysate as well as cell medium of the cultured NPCs.

To this end, cells were cultured in 6-well plates in the above-stated conditions and split until P4 was achieved. The cell medium was separated and stored at 4 °C. The cells were placed on ice and washed with ice-cold PBS three times. Next, 1 mL of RIPA buffer (Abcam, Cambridge, UK) was applied to each well, and adherent cells were scraped off the bottom of the wells and transferred to centrifuge tubes. Centrifugation was maintained for 30 min at 4 °C, the pellet was discarded, and the supernatant was stored at 4 °C.

For blotting of supernatant and cell lysate, we used precast gels (BIO-RAD, Hercules, CA, USA) loaded the running wells with the protein samples and a WesternFroxx all-in-one protein marker 15–200 kDA 10 bands ladder for protein reference (Biofroxx, Heidelberg, Germany). For positive control, we used 1 µg/lane of recombinant human Shh (R&D Systems, Minnesota, MN, USA). We applied 200 V for 40 min. Proteins were transferred onto polyvinylidene difluoride (PVDF) membranes, and the membranes were then incubated in blocking solution containing 5% milk powder (Sigma-Aldrich, Burlington, MA, USA) for 1 h at room temperature. Following blocking, we applied an anti-Shh antibody (1:1000, Origene, Rockville, MD, USA) and incubated the membranes at 4 °C overnight.

After washing the membranes with TBS-T (Tris-Buffered Saline-Tween 20) three times, we incubated the secondary HRP conjugated anti-mouse antibody (1:3000; BIO-RAD, Hercules, CA, USA) for 1 h. After washing three times with TBS-T, we applied clarity western ECL luminol (BIO-RAD, Hercules, CA, USA) and placed the membranes in a darkroom box. For image taking, the camera exposure was set to 2 min.

### 2.6. Statistical Analysis

Normality assumption was evaluated before all parametric analyses using Shapiro-Wilk normality tests. A unilateral variance analysis (ANOVA) followed by a posthoc Tukey-HSD test was performed for the statistical comparison of means between multiple groups. All results are given as mean ± standard error of the mean (SEM), and a *p*-value of *p* < 0.05 was considered significant. All statistical analyses were performed using the software Prism (GraphPad Software, San Diego, CA, USA) in version 8.

## 3. Results

### 3.1. The Survival of NPCs Is Influenced by Shh-Signaling

We used CCK-8 viable cell quantification to measure the survival of NPCs concerning stimulation or blockage of the Shh-pathway under normal and inflammatory growing conditions.

**Figure 2:**
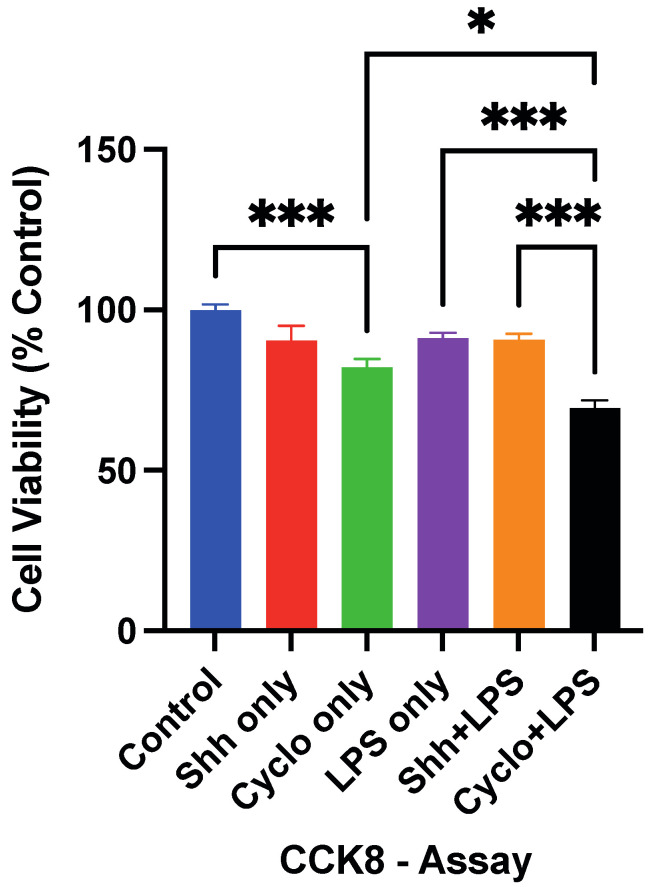
Exogenously administered Shh does not lead to significant changes in the survival of NPCs under normal (Control vs. Shh only; *p* = 0.1532) and inflammatory (LPS only vs. Shh+LPS; *p* > 0.9999) growing conditions in the CCK-8 viable cell quantification after 24 h. However, blockage of the Shh-pathway via administration of Cyclopamine decreases NPC-survival under normal (Control vs. Cyclo only; *p* = 0.0007) and inflammatory (LPS only vs. Cyclo+LPS: *p* < 0.001 and Cyclo+LPS vs. Shh+LPS; *p* < 0.001) growing conditions. Experiments performed in triplicate (*n* = 3), data expressed in percentage of the control group and presented as mean ± SEM, one-way-ANOVAs with post hoc Tukey-HSD tests performed for statistical analysis (* *p* < 0.05; *** *p* < 0.001).

Under normal growing conditions, NPCs in the Shh only group did not display a significant survival change with the Shh-treatment compared to the untreated cells in the control group (90.39 ± 4.71% vs. 100 ± 1.63%; *p* = 0.1532). Yet, inhibition of the Shh-pathway with Cyclopamine significantly reduced cell survival (82.04 ± 2.68%) compared to the untreated control group (*p* = 0.0007; Figure 2). When put in direct comparison, Cyclopamine-treated NPCs showed a slight but not significant trend towards decreased cell survival compared to the Shh-treatment (*p* = 0.1024).

After incubation with LPS, NPCs in the LPS only group did not exhibit a significant difference in terms of survival (91.20 ± 1.57%) compared to untreated cells (*p* = 0.2248). Similarly, cells in the Shh+LPS group did not show a relevant difference in survival (90.70 ± 1.86%) compared to the LPS only group (*p* > 0.9999).

On the other hand, we found that Cyclopamine- and LPS-treated NPCs survived significantly less often (69.39 ± 2.42%) than cells that were incubated with LPS only (*p* < 0.001) and cells receiving the Shh- and LPS-treatment (*p* < 0.001; Figure 2). In addition, Cyclo+LPS treated cells showed significantly less viable cells compared to Cyclo only treated cells (69.39 ± 2.42% vs. 82.04 ± 2.68; *p* = 0.026).

These results may suggest that stimulation of the Shh-pathway via the Shh-ligand does not have a relevant effect on NPC-survival neither under standard nor under inflammatory growing conditions. Nevertheless, the inhibition of the Shh-signaling via Cyclopamine might be associated with adverse survival effects for NPCs, which shows to be even more severe in inflammatory conditions. This might implicate that the activation of the Shh-pathway is important for NPC survival in an inflammatory setting.

### 3.2. Stimulation of the Shh-Pathway Enhances the Proliferation of NPCs

**Figure 3:**
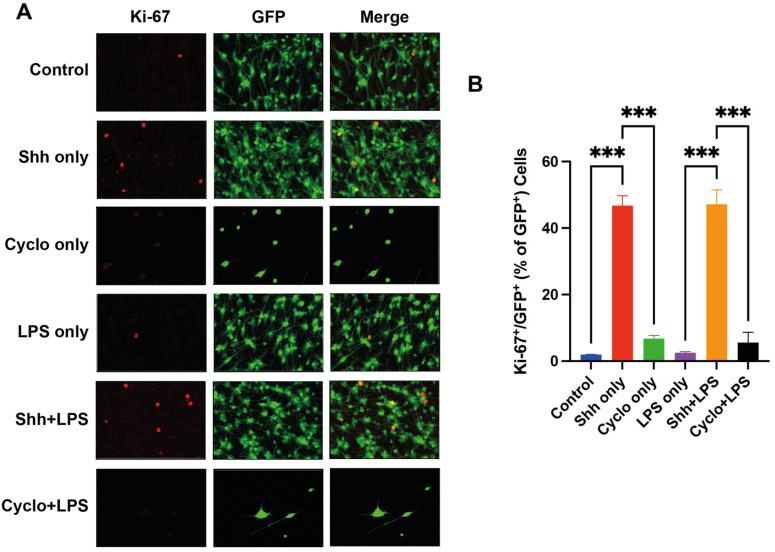
Assessment of the NPC-proliferation rate by quantification of Ki-67^+^/GFP^+^ cells. (**A**) Immunofluorescence staining for Ki-67 (red), GFP^+^ (green) as well as Ki-67^+^/GFP^+^ cells. (**B**) NPC-proliferation rates are significantly increased with the Shh-treatment under normal (Control vs. Shh only; *p* < 0.001) and inflammatory (LPS only vs. Shh+LPS; *p* < 0.001) growing conditions. Experiments were performed in triplicate (*n* = 3), data presented as mean ± SEM, one-way-ANOVAs with post hoc Tukey-HSD tests performed for statistical analysis (*** *p* < 0.001).

We examined the potential of Shh to activate the cell cycle and induce the proliferation of NPCs (GFP^+^ cells) under normal growing and inflammatory stress conditions by quantification of the proliferation marker Ki-67 (Figure 3A).

Under normal cell culture conditions, the rate of Ki-67 positive cells, and thus, the rate of proliferating NPCs was significantly increased with the Shh-treatment compared to the untreated control group (46.78 ± 2.94% vs. 1.99 ± 0.01%; *p* < 0.001). When the Shh-pathway was inhibited with Cyclopamine, the rate of Ki-67 positive NPCs was not significantly affected in the Cyclopamine only group (6.76 ± 0.95%) compared to the control group (*p* = 0.755) but significantly decreased compared to the Shh only group (*p* < 0.001).

Under inflammatory stress induced by LPS, NPCs in the LPS only group showed no statistical difference in the Ki-67 positive cell rate (2.52 ± 0.34%) compared to the control group with cells under normal growing conditions (*p* > 0.999). When NPCs under inflammatory stress were treated with Shh, the rate of Ki-67 positive cells (47.20 ± 4.28%) was significantly increased compared to the LPS only group (*p* < 0.001). In contrast, inhibition of the Shh-pathway in NPCs under inflammatory stress in the Cyclo+LPS group (5.58 ± 3.1%) seemed to increase the proliferation rate compared to the LPS only group, but this difference remained insignificant (*p* = 0.948). Similarly, when compared to the Shh+LPS group, NPCs under inflammatory stress and with Shh-inhibition via Cyclopamine showed a significantly decreased rate of Ki-67 positive cells (*p* < 0.001) and thus reduced proliferation (Figure 3B).

Taken together, our findings suggest that the Shh-signaling might be modulating the proliferation rate of NPCs already under normal growing conditions and under inflammatory stress induced by LPS in vitro.

### 3.3. The Oligodendroglial Differentiation of NPCs Is Not Affected by Shh-Pathway Modulation

**Figure 4:**
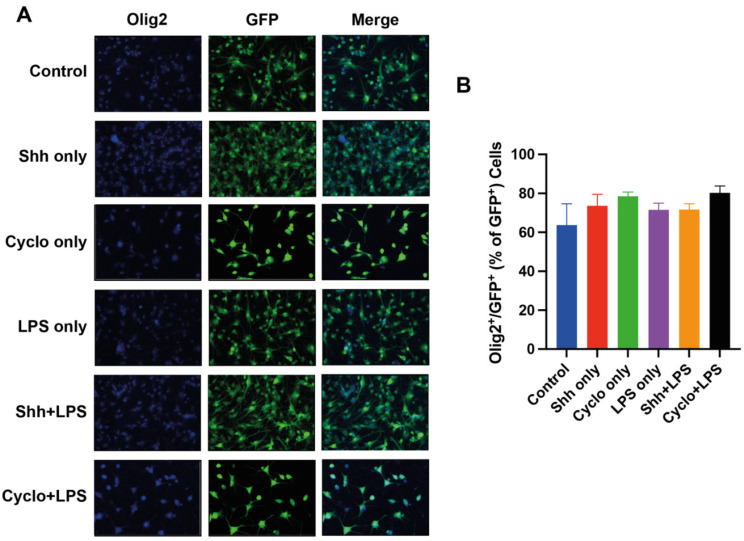
Assessment of the oligodendroglial NPC-differentiation rate by quantification of Olig2^+^/GFP^+^ cells. (**A**) Immunofluorescence staining for Olig2 (blue), GFP^+^ (green) NPCs and Olig2^+^/GFP^+^ (**B**) The rate of oligodendroglial NPC-differentiation (Olig2^+^/GFP^+^ cells per GFP^+^ cells) is not significantly affected by stimulation or blockage of the Shh-pathway under normal and inflammatory growing conditions. Experiments performed in triplicate (*n* = 3), data presented as mean ± SEM, one-way-ANOVAs with post hoc Tukey-HSD tests performed for statistical analysis.

To understand the role of the Shh-pathway in oligodendroglial differentiation of NPCs, we quantified the co-expression of Olig2/GFP to assess their differentiation into mature oligodendrocytes under normal growing inflammatory stress conditions (Figure 4A).

Surprisingly, the Shh-treatment did not significantly increase Olig2^+^/GFP^+^ oligodendrocytes per GFP^+^ cells and thus the oligodendroglial differentiation rate (73.62 ± 5.89%) compared to untreated control cells (63.72 ± 10.91%; *p* = 0.8090). Furthermore, compared to the control group, the inhibition of the Shh-pathway with Cyclopamine (78.52 ± 2.16%) did not significantly decrease but rather increased oligodendroglial differentiation of NPCs (*p* = 0.4699). Thus, the difference between the Shh-treated and Cyclopamine-treated NPCs in terms of oligodendroglial differentiation remained insignificant as well (*p* = 0.987).

Similarly, NPCs under inflammatory stress with LPS (71.53 ± 3.40%) did neither exhibit significantly more oligodendroglial differentiation with the addition of Shh (71.71 ± 2.94%; *p* > 0.999) nor significantly less oligodendroglial differentiation with the addition of Cyclopamine (80.31 ± 3.48; *p* = 0.872). Even the difference in terms of Olig2^+^/GFP^+^ cells per GFP^+^ cells between the Shh+LPS and the Cyclo+LPS group remained unsignificant (*p* = 0.881). Of note, LPS-treated cells did not display a statistical difference in oligodendroglial differentiation compared to untreated cells (71.53 ± 3.40% vs. 63.72 ± 10.91%; *p* = 0.9163) (Figure 4B).

To concise, in our experiment, oligodendroglial differentiation was affected by neither stimulation nor blockage of the Shh-pathway under normal and inflammatory growing conditions.

### 3.4. The Shh-Pathway Is Associated with Pro-Neuronal Differentiation of NPCs

**Figure 5:**
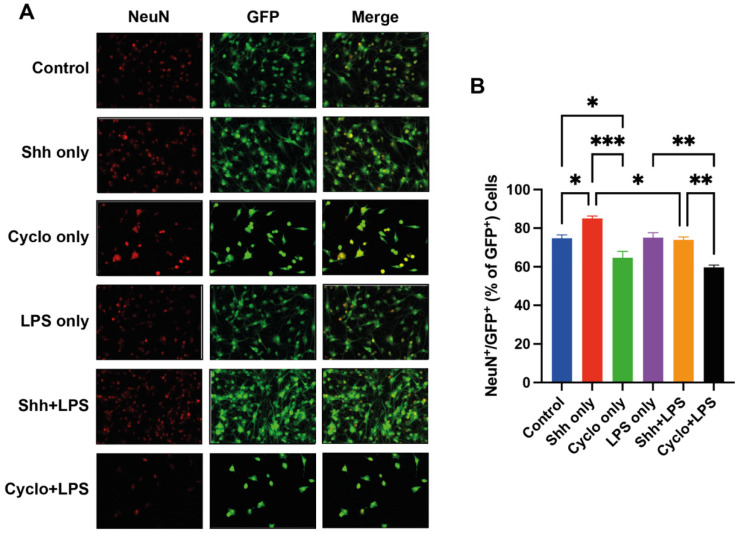
Assessment of the neuronal NPC-differentiation rate by quantification of NeuN^+^/GFP^+^ cells. (**A**) Immunofluorescence staining for NeuN (red), GFP^+^ (green) NPCs, as well as NeuN^+^/GFP^+^ NPC-derived neurons. (**B**) The rate of neuronal NPC-differentiation (NeuN^+^/GFP^+^ cells per GFP^+^ cells) is significantly increased by stimulation of the Shh-pathway under normal growing conditions (Control vs. Shh only; *p* = 0.0370) and significantly decreased by blockage of the Shh-pathway under both, standard (Control vs. Cyclo only; *p* = 0.04) and inflammatory (LPS only vs. Cyclo+LPS; *p* = 0.001) growing conditions. Of note, the pro-neuronal differentiative effect of Shh seems to be decreased under influence of LPS (Shh only vs. Shh+LPS; *p* = 0.0224) Experiments performed in triplicate (*n* = 3), data presented as mean ± SEM, one-way-ANOVAs with post hoc Tukey-HSD tests performed for statistical analysis (* *p* < 0.05, ** *p* < 0.01, *** *p* < 0.001).

Associations between the Shh-pathway and neuronal differentiation of NPCs under normal growing and inflammatory stress conditions were assessed by quantifying the co-expression of NeuN/GFP, indicating NPC-derived mature neurons (Figure 5A).

Interestingly, under normal growing conditions, the percentage of NeuN^+^/GFP^+^ cells and thus NPC-derived neurons was significantly increased with the Shh-treatment (85.03 ± 2.22%), compared to the untreated cells (74.8 ± 3.02%; *p* = 0.0370) and the Cyclopamine-treated cells (64.72 ± 5.682%; *p* = 0.002). Furthermore, blockage of the Shh-pathway in the Cyclo only group led to a significant decrease of neuronal differentiation compared to the control group (*p* = 0.04).

However, contrary to our expectations, this association of the Shh-pathway with the neuronal differentiation of NPCs did not show the same pattern under inflammatory growing conditions: NeuN^+^/GFP^+^ cells were not significantly increased in the Shh+LPS treatment compared to the LPS only group (73.94 ± 1.507% vs. 75.11 ± 4.287%; *p* = 0.9982). Of note, when compared to Shh only, Shh+LPS displayed significantly less differentiated neurons (73.94 ± 1.51% vs 85.03 ± 1.28%; *p* = 0.0224), indicating that LPS might impede otherwise Shh-mediated pro-neuronal differentiation.

Nevertheless, blockage of the Shh-pathway in the Cyclo+LPS group (59.66 ± 01.944%) led to a significant decrease of neuronal NPC-differentiation compared to the Shh+LPS (*p* = 0.003) and the LPS only group (*p* = 0.001) (Figure 5B).

Of note, compared to the control group, LPS-treated cells did not show a difference in neuronal differentiation (74.80 ± 1.745% vs. 75.11 ± 4.287%; *p* > 0.9999).

**Figure 6:**
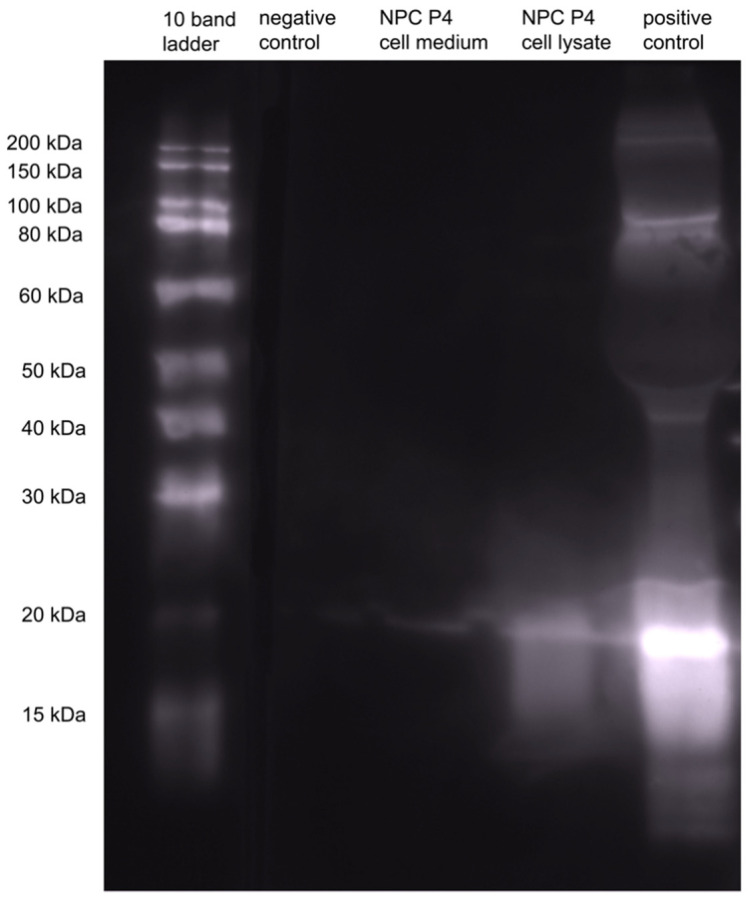
To examine endogenous Shh production by NPCs we performed a qualitative western blot analysis of NPC medium and lysate (P4). Shh is present in the lysate and the medium of untreated NPCs, proving endogenous Shh production.

These findings suggest that the Shh-pathway affects neuronal differentiation of NPCs. Furthermore, decreased neuronal differentiation by inhibition of the Shh-pathway implicates the presence of Shh in the native NPC milieu. To support this finding, we assessed the endogenous Shh production of NPCs under normal growing conditions in vitro and were able to show the presence of Shh in cell lysate as well as cell culture medium in western blot analyses (Figure 6).

### 3.5. Blockage of the Shh-Pathway Is Related to Astroglial Differentiation of NPCs

**Figure 7:**
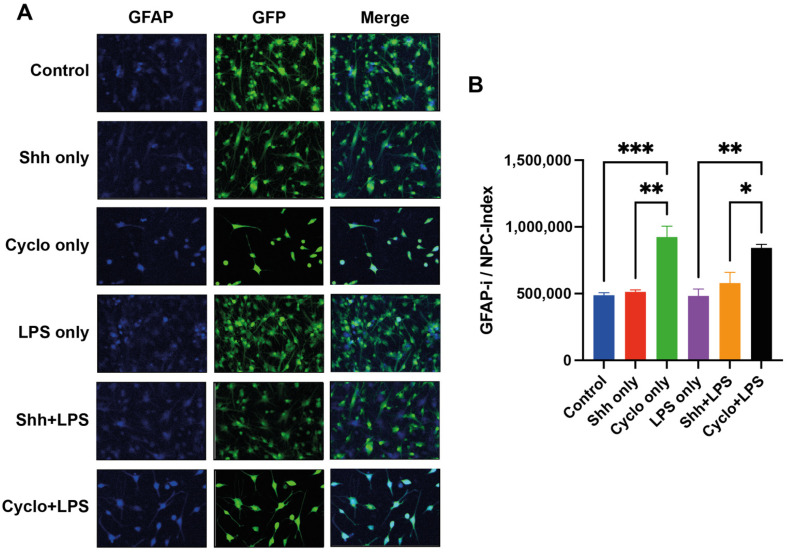
Assessment of the astroglial differentiation of NPCs by calculation of the GFAP-immunointensity (GFAP-i; pixel intensity (pi)/mm^2^) per GFP^+^ cells (GFAP-i/NPC index). (**A**) Immunofluorescence staining for GFAP (blue) indicating glial differentiation, GFP^+^, and GFAP^+^/GFP^+^ cells. (**B**) Under normal growing conditions, but also under inflammatory stress, Cyclopamine-treatment and thus blockage of the Shh-pathway results in a significant increase of the GFAP-i/NPC index, and therefore, significantly more astrocytes compared to untreated cells (Cyclo only vs. control; *p* = 0.0009 and Cyclo+LPS vs. LPS only; *p* = 0.004, respectively) and Shh-treated cells (Cyclo only vs. Shh only; *p* = 0.002 and Cyclo+LPS vs. Shh+LPS; *p* = 0.038, respectively). Experiments performed in triplicate (*n* = 3), data presented as mean ± SEM, one-way-ANOVAs with post hoc Tukey-HSD tests performed for statistical analysis (* *p* < 0.05, ** *p* < 0.01, *** *p* < 0.001).

To further assess the differentiation of NPCs towards the astroglial lineage in dependence of Shh-pathway modulation under normal and inflammatory growing conditions, we used GFAP, a marker for astrocytes, and calculated the GFAP-immunointensity (pi/mm^2^) in relation to the number of GFP^+^ cells (GFAP-i/NPC index) (Figure 7A).

Here, we found that NPCs treated with Cyclopamine (924,538 ± 80,511 pi/mm^2^/NPC) had a higher GFAP-i/NPC index and thus differentiated significantly more towards the astroglial lineage compared to untreated cells (487,971 ± 18,301 pi/mm^2^/NPC; *p* = 0.0009) and Shh-treated cells (512,925 ± 14,845 pi/mm^2^/NPC; *p* = 0.002). However, stimulation of the Shh-pathway with Shh did not affect the GFAP-i/NPC index compared to the untreated control group (*p* = 0.999).

Similarly, under inflammatory growing conditions, NPCs in the Shh+LPS group did not exhibit significant changes in their GFAP-i/NPC index compared to the LPS only group (578,230 ± 79,961 pi/mm^2^/NPC vs. 482,712 ± 52,147 pi/mm^2^/NPC; *p* = 0.786), while the differentiation into astrocytes was significantly increased in the Cyclo+LPS group (843,075 ± 26,651 pi/mm^2^/NPC) compared to the LPS only (*p* = 0.004) and Shh+LPS (*p* = 0.038) groups.

LPS treated cells did not display a statistical difference compared to untreated cells (487,971 ± 18,301 pi/mm^2^/NPC vs. 482,712 ± 52,147 pi/mm^2^/NPC; *p* > 0.9999).

These results may indicate that rather the deprivation but not the stimulation of Shh-signaling impacts astroglial differentiation of NPCs under normal and inflammatory growing conditions (Figure 7B).

### 3.6. Deprivation of Shh-Signaling in NPCs Is Associated with Less Differentiation

**Figure 8:**
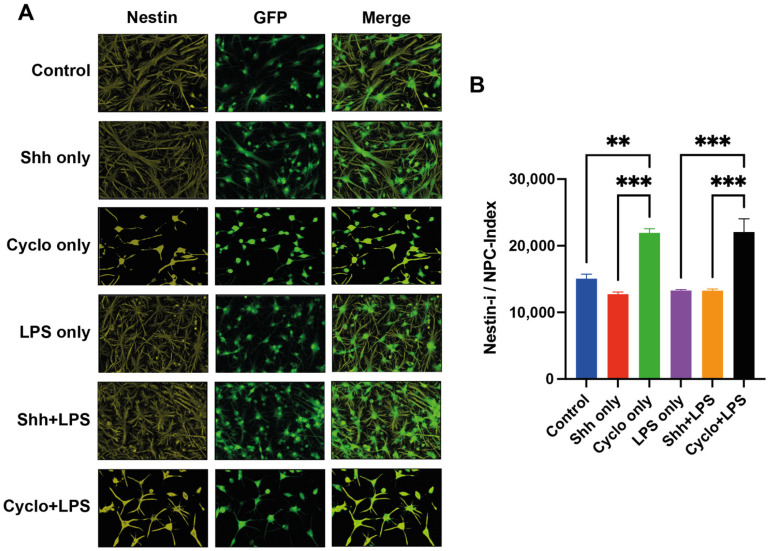
Assessment of NPCs remaining in an undifferentiated state by calculation of the Nestin-immunointensity (Nestin-i; pixel intensity (pi)/mm^2^) per GFP+ cells (Nestin-i/NPC index). (**A**) Immunofluorescence staining for Nestin (yellow), GFP+ (green) NPCs as well as Nestin+/GFP+ immature NPCs. (**B**) Under normal growing conditions but also inflammatory stress, Cyclopamine-treatment and thus blockage of the Shh-pathway results in a significant increase of the Nestin-i/NPC index and thus significantly more undifferentiated NPCs compared to untreated cells (Cyclo only vs. control; *p* = 0.0017 and Cyclo+LPS vs. LPS only; *p* = 0.0002, respectively) and Shh-treated cells (Cyclo only vs. Shh only; *p* < 0.001 and Cyclo+LPS vs. Shh+LPS; *p* < 0.001, respectively). Experiments performed in triplicate (*n* = 3), data presented as mean ± SEM, one-way-ANOVAs with post hoc Tukey-HSD tests performed for statistical analysis (** *p* < 0.01, *** *p* < 0.001).

We quantified the immunointensity (pi/mm^2^) of Nestin (Nestin-i), a marker for undifferentiated NPCs, in relation to the number of GFP^+^ cells (Nestin-i/NPC index), to evaluate the impact of Shh-modulation on NPC-differentiation in general under normal and inflammatory growing conditions (Figure 8A).

Interestingly, with blockage of the Shh-pathway via Cyclopamine, significantly more NPCs remained in an undifferentiated state with a higher Nestin-i/NPC index (21,939.1 ± 622.1 pi/mm^2^/NPC) compared to untreated cells (15,062.8 ± 679.6 pi/mm^2^/NPC; *p* = 0.0017) and Shh-treated cells (12,725.6 ± 336.5 pi/mm^2^/NPC; *p* < 0.001) under normal growing conditions. On the other hand, stimulation of the Shh-pathway in the Shh only group reduced the Nestin-i/NPC index compared to the control group, without reaching a statistically significant difference (*p* = 0.480, Figure 8B).

Under inflammatory stress induced by LPS, inhibition of Shh-signaling in the Cyclo+LPS group (22,049.4 ± 1955.24 pi/mm^2^/NPC) also significantly increased the Nestin-i/NPC index and thus the number of undifferentiated NPCs compared to cells in the LPS only group (13,283.9 ± 144.9; *p* = 0.0002) and the Shh+LPS group (13,270.9 ± 238.7 pi/mm^2^/NPC; *p* < 0.001). Like the findings under normal growing conditions, treatment with exogenous Shh while under inflammatory stress did not significantly influence the general differentiation of NPCs (*p* > 0.999).

LPS treated cells did not display a statistically significant difference in comparison to the control group (*p* = 0.7283).

Together, inhibition of the Shh-signaling under normal and inflammatory growing conditions might prevent NPCs from differentiation, leading to more undifferentiated cells.

## 4. Discussions

Spinal cord injury is a devastating diagnosis that severely affects patients, relatives, and caregivers. The global incidence of spinal cord injury has been estimated at 92 to 2460 per 100,000 inhabitants worldwide [22]. Even though a growing number of patients benefit from improved acute care management and therapy, especially those with severe injuries are left only with years of physical and occupational rehabilitation to mitigate deficits and train available resources. This group of patients is threatened to lose their ability to live a self-sustaining and autonomous life [17,23,24,25].

Decades of SCI injury research and clinical trials have provided a profound understanding of complex pathophysiological processes, such as neuronal inflammation and possible therapeutic targets. However, so far, all efforts have failed to discover feasible treatment options for those patients who are affected the most [18,25,26,27,28,29,30]. Therefore, it is important to investigate further approaches to protect and regenerate neuronal and glial structures after injury.

The Shh-pathway has been under scientific investigation since its discovery in 1980 [31]. Its importance in the embryonal developmental process and tumorigenesis has been intensely researched and published since then–but still, discoveries in the context of the Shh-pathway are being made. As such, recent findings are ascribing Shh a crucial role in the neuroregeneration after CNS injuries [32,33,34,35]. New evidence even suggests that the activation of the Shh-pathway may be necessary to enable MSC-derived exosome mediated SCI repair [36].

Another mechanism by which the Shh-pathway might exert such neuroregenerative capabilities could be its association with the proliferation and differentiation of endogenous NPCs, even in the adult CNS [4,37]. In the context of growing evidence for the relevance of endogenous NPCs in response to CNS injuries [10,38,39], Shh might thus have the potential to improve endogenous neuroregeneration by modulation of NPC niches in, e.g., the spinal cord. Moreover, given the recent interest in stem cell transplantation as a therapeutic option after SCI [26,40,41,42], whereby NPCs play a dominant role [13,21,43,44], a better understanding of the interactions between Shh and the proliferation/differentiation of NPCs might have implications for such cellular-based treatments as well. Of note, a common problem of current NPC-transplantation strategies is the low survival of the cell graft which might be improved by synergistic Shh-treatment [45,46].

Therefore, our current study aimed to assess how NPCs react to stimulation or blockage of the Shh-pathway in terms of survival, proliferation, and differentiation in vitro, preferably in an environment that stimulates the hostile and inflammatory environment of a spinal cord injury.

Unfortunately, reproduction of such an injury in vitro is difficult to achieve because various factors and cells contribute to different injury stages [17,47,48]. Several published methods to reproduce different types of neuronal injury in vitro have been described: Deprivation of glucose and oxygen and consequent reoxygenation [49,50,51] have been used to mimic stroke or other hypoperfusion injuries. Unfortunately, this method seems to lack a certain amount of proinflammatory components. Another model is based on oxidative injury using hydrogen peroxide to damage cells, leaving cell debris in the medium and mimicking acute post-injury stages. However, based on our own experience (data not shown), this method might cause severe and uncontrollable cell death, which complicates consistent data replicability when experiments are reproduced. On the other hand, pro-inflammatory processes are key events, especially in the subacute phase after all CNS injuries [52,53]. Therefore, for our current study, we chose to use LPS to activate inflammatory cascades that impact NPCs via secreted molecules from glial precursor cells [54].

On a side note, the inflammatory growing conditions induced by the LPS-treatment did not lead to statistically significant differences compared to the normally incubated NPCs in the control group in our study. In comparison, Grasseli et al. described an LPS-induced increase in proliferation and pro-neuronal and pro-oligodendroglial differentiation in human neural stem cells by activating Toll-like receptor 4 on neural cells [55]. On the other hand, by activating TLR2, another LPS binding receptor, Okun et al. observed a decrease in mouse-derived NPC proliferation [56]. Neuronal and glial TLR activation seem to have conflicting effects on neurogenesis, neuroprotection, and neuroinflammation [57,58]. The different responses to TLR activation seem also to be affected by other factors, e.g., the type of injury, the type, and maturity of neurons, the presence of glial cells [59], or the type of experimental setting [57]. Further data collection would be needed to understand the complexity of LPS induced TLR activation and its effects on neurons in vitro.

In a Western Blot analysis, we were able to demonstrate the presence of Shh in untreated NPC cultures, in cell medium, as well as supernatant (Figure 6), proving that a basal level of Shh expression and secretion is being perpetuated autonomously by NPCs. Shh expression and secretion by glial cells [33] and even by mature neurons, e.g., Purkinje cells and pyramidal cells [60,61,62] has been described before, yet the expression of Shh in NPC cultures has not been described yet. We found that additional stimulation with Shh did not increase the survival of SVZ-derived NPCs in vitro, neither under normal nor under inflammatory growing conditions. On the other hand, we could observe a decrease of NPC-survival by the inhibition of the Shh-pathway via the blockage of the Shh-receptor Ptch with Cyclopamine, suggesting that the above-mentioned basal level of Shh-activation is vital.

Cyclopamine selectively binds and blocks Smoothened, a transmembrane protein and the first signal protein in the Shh-induced Ptch-Smo-Gli-cascade. It can thus be conceivably hypothesized, that while excess activation of the Shh-pathway has no positive effect on NPC-survival, neither under normal nor inflammatory growing conditions, inhibition of that same pathway may aid pro-apoptotic processes and increase cell vulnerability. This is following Cheng et al., who reported that blockage of the Shh-pathway with Cyclopamine resulted in significant cell death after oxygen-glucose deprivation/reoxygenation injury (OGD/R) compared to untreated cells [51].

The relevant biochemical processes by which the Shh-pathway is linked to cellular survival remain ill-defined. It is assumed that complex interaction between Shh-key effector protein Gli1 and other signaling pathways conclude into a pro-survival effect. As an example, Nye et al. described the interplay between Gli1 and TGFβ-associated transcriptional factor SMAD4 as being vital to transcription of BCL2 and consequently to cell survival [63,64]. Of note, in our experiment, the difference in NPC-survival with Shh-signaling inhibition was more distinct in the cells under inflammatory growing conditions, suggesting a stronger dependency on Shh-dependent basal activation when cells are under inflammatory stress. In accordance with our results, previous evidence has highlighted the role of Shh-activation in neuronal stem cell and stem cell-like cell cultures [65,66], even in hypoxic stress models by incubation with 1% O_2_ [67] or in OGD/R injury models [51,66], displaying positive aspects of Shh for cell survival.

On the other hand, the proliferation of NPCs in our current experiment was strongly improved by stimulation with Shh, both in a normal and inflammatory environment. This concurs well with previous findings [51,68,69]. Therefore, our results may not only indicate the consistency of the notion that Shh is a potent proliferation agent for NPCs in a hostile environment but provide evidence that it can exert similar effects on NPCs that are already under optimal growing conditions.

To further understand how Shh-signaling affects the differentiation patterns of SVZ-derived NPCs in a hostile, inflammatory stress environment, we stained the cells for NeuN (neurons), Olig2 (oligodendrocytes), Nestin (undifferentiated NPCs), and GFAP (astrocytes) after Shh-pathway stimulation or blockage.

Interestingly, only the rate of neuronal differentiation was significantly increased after administration of Shh to the NPCs and only while the cells were under normal growing conditions. This evidence aligns with the notion that activation of the Shh-pathway results in pro-neuronal maturation [68,70]. In contrast, in an inflammatory environment, Shh could not increase the NPC-differentiation towards the neuronal lineage. Compared to Shh only, Shh/LPS co-treated NPCs even displayed attenuated pro-neuronal differentiation, although differences in other differentiation markers remained insignificant. These results substantiate the notion that inflammatory damage attenuates pro-neuronal differentiation and are in line with previous results [71,72]. For example, Mao et al. described an increase of Doublecortin (DCX) positive cells, a typical early neuronal marker, after affecting neuroinflammatory cascades by inhibiting the JAK-STAT pathway using overexpression of miR-17-92 [73].

Furthermore, blockage of the Shh-pathway via Cyclopamine significantly reduced the neuronal differentiation of NPCs under normal conditions and inflammatory stress, adding further evidence to the importance of Shh-pathway activity for neuronal differentiation [65,68,70]. At the same time, the treatment with Cyclopamine led to a noticeable increase of undifferentiated NPCs and NPC-derived astrocytes independent from the respective growing conditions, indicating that the inhibition of the Shh-pathway might pave the way to less general and further astrocyte differentiation of NPCs. These results are in line with previous evidence, e.g., Lee et al., who found that human embryonic stem cells treated with Cyclopamine significantly showed more Nestin^+^ cells, and subsequent culturing in the human astrocyte medium showed a significantly higher cell count of GFAP^+^ cells compared to untreated cells [74]. Of note, in our experiment, Shh-pathway modulation did not affect the oligodendroglial differentiation of NPCs. Different and conflicting effects of the Shh-pathway on proliferation and development of oligodendrocytes and oligodendroglial precursor cells (OPC) have previously been described [75,76,77]. These diverging notions are likely due to the complexity of the Shh-pathway and its dependence on other cell-fate deciding proteins, e.g., Fibroblast Growth Factor (FGF), Wnt-1, or Bone Morphogenic Protein (BMP).

Although several in vitro as well as in vivo studies on Shh-pathway modulation support the above-stated evidence, others did come to different conclusions: To put it into a different perspective, Araujo et al. described an increase in proliferation and differentiation of GFAP^+^ astrocytes in telencephalon cell cultures when treated with Shh [78]. At the same time, Hill et al. commented on various instances in which Shh-mediated reactive astrogliosis in the adult brain mitigates further brain injury [79]. Furthermore, an Shh-mediated decrease in NeuN^+^ neurons in favor of undifferentiated granule cell precursors was described in Wechsler-Reya and Scott’s study using cerebellar cell cultures [80]. These different conclusions do not necessarily conflict but emphasize the complexity and difficulty of assessing the Shh-pathway in different environments, cell maturity, and cell types of the CNS, indicating that further research will be needed to develop a broader understanding.

Nevertheless, our current work substantiates former assumptions. It provides more evidence that the stimulation of the Shh-pathway might mediate the proliferation and neuronal differentiation potential of NPCs in vitro, even in an inflammatory stress environment mimicking the subacute phase after CNS injury. At the same time, our results indicate that a reduction of Shh-pathway activation by blockage with Cyclopamine might be associated with reduced NPC-survival, reduced general differentiation and increased astroglial differentiation. Overall, the Shh-pathway might, therefore, be an exciting target to enhance endogenous, NPC-mediated, or even exogenous, NPC-graft related neuroregeneration after SCI with currently minimal treatment options. Beforehand, further research is needed to unveil the full potential of the Shh-pathway and its interactions in the context of CNS injuries in vitro and more importantly in vivo.

## Figures and Tables

**Table 1:** Outline of treatment groups.

Treatment 1	Treatment 2	Group Name
Untreated	Shh	Shh only
	Cyclopamine	Cyclopamine only
	none	Control
LPS	Shh	Shh+LPS
	Cyclopamine	Cyclopamine+LPS
	none	LPS only

## Data Availability

The data presented in this study are available on reasonable request from the corresponding author.
